# Comparison of radiography and computed tomography for identification of third metacarpal structural change and associated assessment of condylar stress fracture risk in Thoroughbred racehorses

**DOI:** 10.1111/evj.14131

**Published:** 2024-08-14

**Authors:** Soroush Irandoust, Linnea M. O'Neil, Christina M. Stevenson, Faith M. Franseen, Pieter H. L. Ramzan, Sarah E. Powell, Sabrina H. Brounts, Samantha J. Loeber, David L. Ergun, R. Chris Whitton, Corinne R. Henak, Peter Muir

**Affiliations:** ^1^ Department of Surgical Sciences University of Wisconsin‐Madison Madison Wisconsin USA; ^2^ Department of Mechanical Engineering University of Wisconsin‐Madison Madison Wisconsin USA; ^3^ Rossdales Veterinary Surgeons Newmarket UK; ^4^ VetCT Cambridge UK; ^5^ Asto CT, 7921 UW Health Ct. Middleton Wisconsin USA; ^6^ Department of Veterinary Clinical Sciences Melbourne Veterinary School, Faculty of Science, University of Melbourne Werribee Victoria Australia; ^7^ Department of Biomedical Engineering University of Wisconsin‐Madison Madison Wisconsin USA; ^8^ Department of Orthopedics & Rehabilitation University of Wisconsin‐Madison Madison Wisconsin USA

**Keywords:** condylar stress fracture, digital radiography, fetlock, horse, racing Thoroughbred, standing computed tomography

## Abstract

**Background:**

Catastrophic injury has a low incidence but leads to the death of many Thoroughbred racehorses.

**Objectives:**

To determine sensitivity, specificity, and reliability for third metacarpal condylar stress fracture risk assessment from digital radiographs (DR) and standing computed tomography (sCT).

**Study design:**

Controlled ex vivo experiment.

**Methods:**

A blinded set of metacarpophalangeal joint DR and sCT images were prepared from 31 Thoroughbreds. Four observers evaluated the condyles and parasagittal grooves (PSG) of the third metacarpal bone for the extent of dense bone and lucency/fissure and assigned a risk assessment grade for condylar stress fracture based on imaging features. Sensitivity and specificity for detection of subchondral structural changes in the condyles and PSG, and for risk assessment for condylar stress fracture were determined by comparison with a reference assessment based on sCT and joint surface examination. Agreement between observers and the reference assessment and reliability between observers were determined. Intra‐observer repeatability was also assessed.

**Results:**

Sensitivity for detection of structural change was lower than specificity for both imaging methods and all observers. For agreement with the reference assessment of structural change, correlation coefficients were generally below 0.5 for DR and 0.49–0.82 for sCT. For horses categorised as normal risk on reference assessment, observer assessment often agreed with the reference. Sensitivity for risk assessment was lower than specificity for all observers. For horses with a reference assessment of high risk of injury, observers generally underestimated risk. Diagnostic sensitivity of risk assessment was improved with sCT imaging, particularly for horses categorised as having elevated risk of injury from the reference assessment. Assessment repeatability and reliability was better with sCT than DR.

**Main limitations:**

The ex vivo study design influenced DR image sets.

**Conclusions:**

Risk assessment through screening with diagnostic imaging is a promising approach to improve injury prevention in racing Thoroughbreds. Knowledge of sensitivity and specificity of fetlock lesion detection provides the critical guidance needed to improve racehorse screening programs. We found improved detection of MC3 subchondral structural change and risk assessment for condylar stress fracture with sCT ex vivo.

## 1 INTRODUCTION

Risk of exercise‐associated mortality and risk of catastrophic injury for Thoroughbred racehorses can be as high as 0.55% of race starts^1^ with a pooled incidence of 0.115% in flat racing.^2^ The regional incidence of third metacarpal/metatarsal (MC3/MT3) condylar stress fractures is variable but can represent 25% of catastrophic injuries in some regions such as California^3^ and is more common in the MC3 bone compared with the MT3 bone.^4^ Progressive accumulation of subchondral fatigue microdamage over time eventually leads to structural failure of the bone.^5^ This highlights the potential benefit of a screening approach to injury prevention, as misdiagnosis of the progressive fatigue damage can lead to catastrophic fractures.^6^ Due to the shape of the distal end of the MC3/MT3 bone, superimposition of the proximal sesamoid bones with the distal MC3/MT3 on radiographs, the density of the bone end, and the minimal clinical signs^7^ often associated with fatigue injury to the distal MC3/MT3, screening to identify racehorses in training with a high imminent risk of condylar stress fracture is challenging. Such early detection is important, because subsequent progression to a complete stress fracture at high speed is associated with risk of mortality, surgical intervention and serious career interruption or curtailment. Athletic performance after surgical treatment of Thoroughbreds with condylar stress fracture can be disappointing because of osteoarthritis, the presence of multiple condylar fractures, fracture comminution, and fractures in adjacent bones.^4^, ^8^, ^9^ There is substantial societal concern regarding the welfare of Thoroughbred racehorses and the problem of catastrophic musculoskeletal injury and associated risk of jockey injury is a particular focus.^10^ Screening the Thoroughbred racehorse fetlock to mitigate the risk of condylar stress fracture and the potential for serious or catastrophic injury is an area of intense interest currently.^10^ However, such screening approaches need to be accurate.

At the population level, achieving substantial reduction in incidence of catastrophic injury continues to be a challenging problem to address, in part because gait changes associated with fetlock parasagittal groove (PSG) subchondral bone injury (SBI) may only be evident at full racing speed and pre‐race veterinary inspection may not reveal concerning clinical signs.^7^, ^11^ Interactions between limb loading from athletic activity and racing surface properties, microdamage accumulation and bone remodelling appear complex. Consequently, there is increasing focus on screening of the individual horse using diagnostic imaging to identify at‐risk horses and more research is needed.^10^ Any effective screening test to prevent an acute potentially catastrophic event should be highly sensitive (few false negatives) for detection of at‐risk horses. Test specificity (few false positives) is also impactful, because the Thoroughbred racehorse industry will not accept a screening test if too many healthy horses are scratched from races. Strategies such as profiling and serial assessment may be required to appropriately identify a subpopulation at greater risk and thereby improve the positive predictive value of any applied diagnostic test.^10^

Very high cyclic loads are transferred from the proximal sesamoid bones to the condyles of the MC3/MT3 during racing.^12^ With race training, isotropic trabecular bone in the distal end of the MC3/MT3 bone becomes remodelled into anisotropic bone with parallel sagittal plates extending proximally from the articular surface that are connected by thin mediolateral struts.^13^, ^14^, ^15^, ^16^ Fracture is initiated through local accumulation of fatigue microdamage arising from calcified cartilage with coalescence into a macroscopic PSG fatigue crack.^5^, ^17^, ^18^, ^19^ Once PSG fatigue damage has compromised the mechanical integrity of the cortical shell of the distal end of the bone, a condylar stress fracture may propagate proximally through the thin medial–lateral struts in the trabecular bone,^17^ although initial propagation may be delayed by focally increased adaptive sclerosis of the distal MC3/MT3 in response to high strain cyclic loading. An associated remodelling response is typical and may be seen as localised bone lysis in the PSG of the subchondral bone radiographically.^6^, ^17^, ^18^, ^19^, ^20^ Subchondral fatigue cracks may also propagate obliquely to form a palmar/plantar osteochondral disease (POD) lesion which occurs primarily within the condyle.^18^ The advanced POD lesion is typically a saucer shaped SBI that is also initiated by accumulation of articular microcracks, initially in the calcified cartilage zone. Although uncommon, condylar fractures that arise more abaxially in the joint surface can be initiated by subchondral fatigue injury associated with a POD lesion.^18^, ^21^, ^22^ POD lesions are not typically evident with DR unless pathologic changes are severe; their role in risk of catastrophic injury is currently considered to be minimal but is not fully determined.^23^, ^24^

Incipient condylar fracture can be diagnosed by planar digital radiography (DR) through identification of altered radiodensity in the PSG region.^6^ However, the reliability of DR for detection of concerning SBI in the PSG that elevates imminent risk of serious injury from condylar stress fracture is unclear. Focal PSG SBI in some horses may not be detectable by DR,^25^ and the consequence of a false negative diagnosis may be catastrophic.^6^ Standing computed tomography (sCT) may enhance identification of fatigue‐induced structural change.^25^, ^26^ However, the usefulness of both modalities is contingent upon equipment, technique, and user interpretation. Currently, there is little published information available to guide clinicians in the application of DR or sCT for identification of Thoroughbred racehorses with concerning PSG SBI associated with increased risk of imminent catastrophic injury from condylar stress fracture. The observation that PSG SBI lesions are much more common in the contralateral limbs of Thoroughbred racehorses with catastrophic condylar stress fracture compared with horses that are not catastrophically injured^27^, ^28^ highlights the need to address this gap in knowledge. In a recent study, 5 of 13 horses with condylar stress fractures had small focal PSG SBI lesions in the contralateral limb, whereas this finding was not observed in any of the eight control horses.^27^ Such lesions often lead to mechanical compromise in affected bones.^29^, ^30^

The flexed dorsopalmar or plantarodorsal radiographic projection is critical for identification of radiographic change reflecting SBI using DR.^6^ Optimal radiographic technique is important for diagnosis of incipient condylar fractures using DR and current best practice uses multiple flexed projections.^31^ The growing application of cross‐sectional imaging methods to the Thoroughbred fetlock over time has encouraged refinement of DR technique and interpretation in recent years^32^ and promises improved screening of Thoroughbred racehorses.^10^

To better understand the role of DR and sCT in detection of incipient condylar fracture, we compared sensitivity and specificity, intra‐observer repeatability, and inter‐observer agreement for detection of fatigue‐induced SBI in the Thoroughbred racehorse fetlock ex vivo using the two imaging methods. We hypothesised that sensitivity and specificity for detection of these structural changes and the fracture risk assessment associated with such changes to subchondral bone would be influenced by imaging method and by the clinical experience of the observer.

## 2 MATERIALS AND METHODS

Because condylar stress fractures are most common in the thoracic limb, ex vivo imaging in this study was limited to the thoracic limb. This helped generate a standardised image set for blinded evaluation by a panel of observers against a gold standard reference assessment that used sCT evaluation and pathological evaluation of the joints for determination of sensitivity and specificity of structural feature detection and risk assessment for condylar stress fracture. For each DR and sCT image set, none of the observers were aware of the identity of the horse. Observers were also blinded to the relationship between DR and sCT images obtained from each horse. Observers assessed the image sets independently.

### 2.1 Sample population of Thoroughbred racehorses

Entire distal limb specimens were obtained from 31 Thoroughbred racehorses that died or were euthanatised for reasons unrelated to the study during athletic training and racing. Horses were euthanatised humanely by a veterinarian at the racetrack because of catastrophic injury. Limbs were transected at the level of the carpus, sealed in plastic bags, and stored at −20°C until needed. Limbs were thawed to room temperature before use.

### 2.2 Fetlock DR

DR imaging used a VetRocket x‐ray generator (HF100/30+ generator, Min‐Xray Inc.) and panel (CDXI‐31, Canon Electron Tubes and Devices Co., Ltd. and Sumito Corporation of the Americas). For each study fetlock, the following carefully collimated radiographic projections were made using a standard protocol^31^ and a positioning jig: (1) Flexed latero‐medial with ~25° of joint flexion; (2) Flexed dorsal–palmar with five projections including dorso‐palmar and flexed dorso7.5° proximal–palmarodistal oblique, flexed dorso15° proximal–palmarodistal oblique, flexed dorso7.5° distal–palmaroproximal oblique, flexed dorso15° distal–palmaroproximal oblique views. Radiographic exposure settings were set at 64 kVp and 4.25 mAs and a focal distance of 91.4 cm (36 inches). Positioning in each image was checked for technical errors^21^, ^31^ before the DICOM images were saved to a network drive. Because of the ex vivo set‐up, each image set was anonymised for evaluation and a screenshot flexed dorsopalmar image of each limb was also prepared to indicate the lateral aspect of the fetlock for the observers.

Blinded DR image sets were evaluated for the extent of dense subchondral bone in the lateral and medial condyles and PSGs (0—absent, 1—mild [<33% of the subregion of the bone end], 2—moderate [33%–66% of the subregion], and 3—severe [>66% of the subregion]). Images were also evaluated for the presence of a subchondral lucency/fissure in the lateral and medial condyles and PSGs (0—absent, 1—poorly defined, no surrounding increased density, 2—poorly defined, with surrounding increased density, 3—well defined, no surrounding increased density, and 4—well defined, with surrounding increased density) (File S1). Each horse was also assigned a risk assessment grade based on structural features from diagnostic imaging (Table 1, File S1). For the purposes of risk assessment screening using diagnostic imaging, ‘overt stress fracture’ was defined as a subchondral structural abnormality associated with sufficient fatigue damage where the observer considered the mechanical properties of the bone compromised based on current knowledge in the field.^6^, ^17^, ^20^, ^25^, ^27^, ^29^, ^30^

**TABLE 1:** Overall assessment of immediate risk of condylar stress fracture in the racing Thoroughbred.

Risk category	Clinical recommendation
Normal	Standard risk. Continue training and racing
Indeterminate	Further imaging recommended
Strong likelihood of stress crack from subchondral fatigue injury	Discontinue fast training and further imaging is recommended
Stress crack present from subchondral fatigue injury	Discontinue training and further imaging recommended to assess significance
Overt stress fracture from fatigue injury	Rehabilitation or surgery is required

### 2.3 Fetlock sCT

Each limb specimen was scanned frozen to enable specimen preservation and to minimise tissue exposure to freeze–thaw cycles. Scanning frozen does not alter CT imaging of osseous structures. sCT imaging was performed at high‐resolution using 0.55 mm contiguous slices using a robotic CT scanning system which has the capability of performing vertical scanning of limb pairs as well as horizontal scanning of the head/neck in the standing sedated horse. The 24‐slice helical Equina scanner (Asto CT Equina) is a fixed installation and has a 240 V single phase 30 A uninterruptable power supply. Scanning was performed using an exposure of 160 kVp and 8 mA at 1 s per 360° revolution using 24 detector rows with a variable helical pitch, typically 0.55. Slice acquisition rate is 36 slices/s with an image acquisition matrix of 1024 × 1024 and a resolution at isocenter of 0.75 mm. Maximum scanning distance is 100 cm vertically at 2 cm/s.^26^ No scout images are needed. Scanning was performed in a horizontal direction with the limbs placed on the positioning table normally used for head scanning in the standing horse. DICOM images were then stored on a network drive. Each image set was anonymised for evaluation using the Horos DICOM viewer and a screenshot transverse image at the level of the MC3 condyles was also prepared to indicate the lateral aspect of the fetlock. Observers adjusted the window width and window level to alter the contrast of the images as needed to optimise assessment of the image set.

Blinded sCT image sets were evaluated by multiplanar reconstruction for increased subchondral bone density in the lateral and medial condyles and PSGs using a 45° oblique dorsoproximal palmarodistal reconstruction perpendicular to the palmar joint surface. The sagittal plane was positioned over the region of interest for grading using an established scale.^33^ The extent of dense subchondral bone in the lateral and medial condyles and PSGs was assessed (0—absent, 1—mild [<33% of the subregion of the bone end], 2—moderate [33%–66% of the subregion], and 3—severe [>66% of the subregion]). Although microdamage cannot be detected in living horses with current imaging methods, the extent of radiographic subchondral bone sclerosis is correlated with microdamage.^34^, ^35^ Also, the thickness of the subchondral plate may be linked to risk of condylar stress fracture.^27^ We considered these important observations in our study design. In each PSG, the subchondral plate thickness (mm) was measured perpendicular to the articular surface of the condyles in the same 45° oblique dorsal proximal to palmar distal reconstruction as a measure of subchondral sclerosis and associated fatigue microdamage (File S1).^27^ Images were also evaluated for the presence of a subchondral lucency/fissure in the lateral and medial condyles and PSGs (0—absent, 1 poorly defined, no surrounding increased density, 2—poorly defined, with surrounding increased density, 3—well defined, no surrounding increased density, and 4—well defined, with surrounding increased density) as with the DR images (File S1). Each horse was also assigned a risk assessment grade based on structural features from diagnostic imaging (Table 1, File S1). Parasagittal area (mm^2^) of the subchondral lucency was measured when identified.^29^

### 2.4 Visual examination of the distal articular surface of the third metacarpal bone and photography

Pathological examination used an established approach.^29^, ^34^ After diagnostic imaging, all non‐cartilaginous soft tissue were removed from each MC3 bone. The shaft was cut with a band saw in the mid‐diaphysis, 16.5 cm (6.5 inches) proximal to the dorsal articular margin of the distal end of the bone. The second and fourth metacarpal bones were removed. The articular surface at the distal end of the bone was photographed and the articular cartilage and any remaining soft tissues were then macerated using 0.15–0.2 M NaOH at 37°C. The solution was changed as needed every 1–3 days. Once the soft tissues were removed, additional photographs of the subchondral bone of the distal articular surface were made.

Severity of fatigue injury to the distal MC3 articular surface in the lateral and medial condyles and PSGs was graded before and after removal of hyaline articular cartilage by maceration (0—absent, 1—mild, 2—moderate, 3—severe). Lesions typically have a parasagittal linear shape in the PSG and a circular shape in the condyle. Features assessed included discoloration of the cartilage or subchondral bone of the palmar joint surface, ulceration/collapse of the cartilage, or presence of subchondral defect.^36^, ^37^, ^38^

### 2.5 Radiographic and pathologic image evaluation

Four observers were used for image assessment with differing clinical experience. A boarded radiologist with extensive experience working with Thoroughbred racehorses (SEP, Observer 1), a boarded large animal surgeon (SHB, Observer 2), and a second boarded radiologist with equine emphasis (SJL, Observer 3) evaluated the blinded DR and sCT image sets. A primary care veterinarian with extensive experience working with Thoroughbred racehorses in training (PHLR, Observer 4) also evaluated the DR image set. In the randomised blinded DR and sCT image sets, images from one Thoroughbred were included five times to determine intra‐observer variation.

Reference (gold standard) interpretation was created by an experienced observer (PM). Review of sCT combined with visual assessment of the joint surface of the distal end of the MC3 bone to further assess fatigue damage to the joint surface was considered the gold standard reference interpretation,^21^ because observation of the joint surface provides direct detailed assessment for the presence of PSG subchondral fatigue cracks. Blinded sCT image sets were evaluated and reference assessment scores for structural change and risk assessment were made using the study assessment form (File S1). Reference assessment of subchondral sclerosis was based on sCT evaluation using a review form for structural change and risk assessment developed by the authors based on their collective extensive experience in the field.^5^, ^6^, ^18^, ^29^, ^30^, ^38^ For limbs from horses with a reference assessment of *overt stress fracture from fatigue injury where rehabilitation or surgery is required* (Table 1), the sCT images of contralateral fetlocks were examined for evidence of stress fracture. Subsequently, joint surface photographs were evaluated from each horse to generate assessment scores for cartilage and SBI. Finally, sCT images were re‐evaluated together with the joint surface photographs to develop an updated gold standard reference interpretation for sCT assessments of SBI and risk assessment score for imminent injury for each horse.

### 2.6 Data analysis

The image sets that were repeatedly scored five times were condensed to a single value for each observer and each modality for inter‐observer comparisons. The median value for the categorical scores, the mean value for continuous measurements, and the majority risk assessment grade were used as the final entry for the repeated horse in the data set for each observer in each modality. Contingency tables were constructed using Prism v10.0 (GraphPad Software) for each imaging method and for each clinician observer to determine sensitivity and specificity for detection of structural changes to distal MC3 subchondral bone and condylar stress fracture risk assessment against the reference assessment. The Fisher's exact test was used to determine significant differences between feature identification from each observer and the reference interpretation for DR imaging, sCT imaging, and the associated risk predictions for each horse. Results were considered significant at *P* < 0.05.

The intra‐class correlation coefficient (ICC) (*r*) statistic was calculated^39^ using MATLAB R2023a (MathWorks) to estimate intra‐observer repeatability, agreement between each observer and the reference assessment, and inter‐observer reliability in rating structural features and condylar stress fracture risk assessment. For interpretation of agreement between each observer and the reference assessment and inter‐observer reliability, <0.5 was considered poor reliability, ≥0.5 to <0.75 was considered moderate reliability, ≥0.75 to <0.9 was considered good reliability, and ≥0.9 was considered excellent reliability.

## 3 RESULTS

### 3.1 Postmortem findings

**FIGURE 1:**
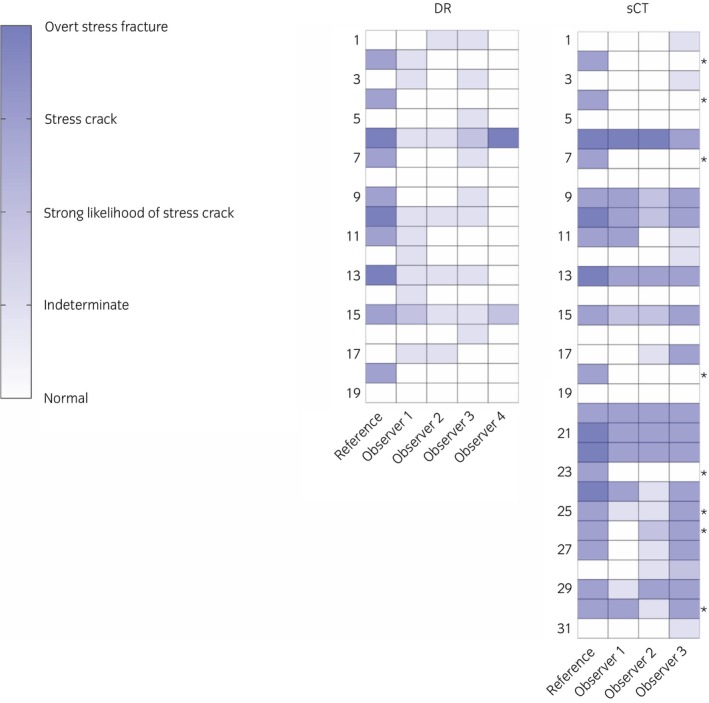
Condylar stress fracture risk assessment from digital radiography (DR) and standing CT (sCT) from a population of racing Thoroughbreds. Reference—Gold standard reference assessment using sCT combined with examination of the joint surface before and after digestion of articular cartilage. For many horses, risk was assessed at a lower level than the reference assessment, particularly for DR. Most horses with elevated fracture risk were identified by multiple sCT observers. * Reference risk assessment for these eight horses was updated from *Normal* based on sCT alone to *Stress crack present from subchondral bone injury* after postmortem evaluation. Horse no. 22 was used for intra‐observer repeatability.

The horses of the study had a range of joint pathologic change from mild to severe (File S2: Figures S1 and S2). After initial evaluations made using sCT, reference risk assessments for eight horses were revised after examination of the articular surface photographs before and after cartilage removal. Risk category from the reference assessment for these eight horses was updated from *Normal* based on sCT alone to *Stress crack present from subchondral bone injury* after postmortem evaluation identified small macroscopic cracks in the PSG (Figure 1). Of these eight horses, five were assessed as *Normal* by all four observers. Assessment of risk was variable for the other three horses across the observers.

### 3.2 Intra‐observer repeatability

Intra‐observer repeatability is reported in Table 2, File S2: Tables S1, and S2. Variation in assessment of structural change was found for all observers with DR (File S2: Table S1). All readings were within one grade, except for one observer with one density grade where the difference was two grades (File S2: Table S1). Lucency/fissure in the PSG with DR imaging was consistently reported as absent (0) by Observer 3. Variation was found in the continuous variables subchondral plate thickness and PSG lucency parasagittal area measured from sCT by all observers as well as the categorical variables (File S2: Table S2). The magnitude of intra‐observer variation for subchondral plate thickness ranged from 6% to 23% across observers and 17% to 157% for PSG lucency parasagittal area.

**TABLE 2:** Intra‐observer repeatability in condylar stress fracture risk assessment determined by digital radiography (DR) and standing computed tomography (sCT) for the racing Thoroughbred with five blinded repeats of the image set.

Risk category	Reference	Observer 1		Observer 2		Observer 3	
		DR	sCT	DR	sCT	DR	sCT
Normal							
Indeterminate		3	1	5		5	
Strong likelihood of stress crack from subchondral fatigue injury		2			1		
Stress crack present from subchondral fatigue injury			4		4		5
Overt stress fracture from fatigue injury	5						

*Note*: Observer 4 reported poor image quality for all five of the DR image sets and did not score any of them. Clinical recommendations for risk assessment are reported in Table 1. The reference assessment was derived from sCT images combined with pathological assessment of the articular surface of the distal end of the third metacarpal bone.

**FIGURE 2:**
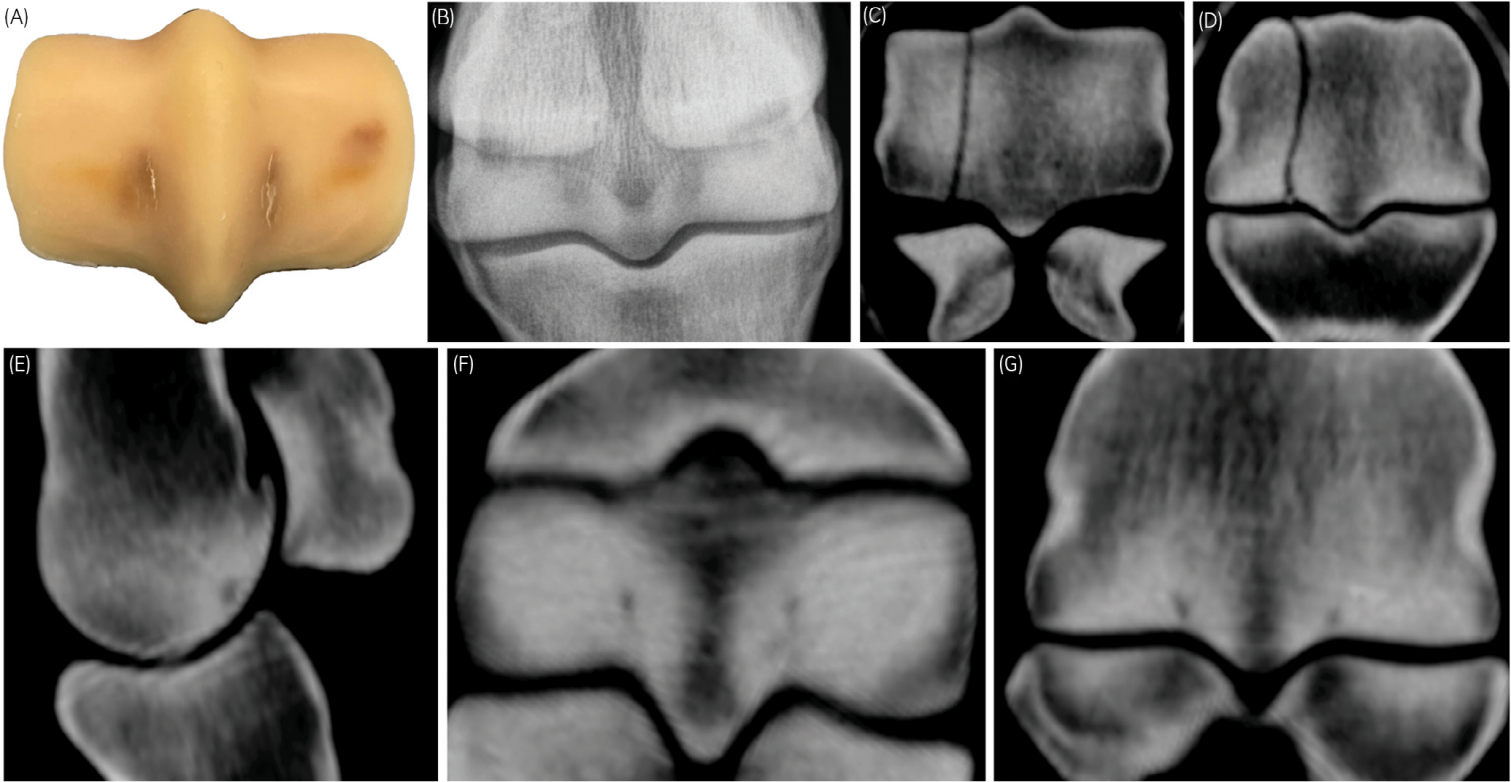
Postmortem (A), digital radiography (DR) (B), and standing computing tomography (sCT) images (C–G) from a racing Thoroughbred with biaxial PSG focal subchondral bone lucencies because of fatigue injury and contralateral condylar stress fracture  (Horse no. 22, Figure 1). The lucencies seen on sagittal (E), transverse (F), and dorsal (G) sCT reconstructions correspond to the linear PSG subchondral fatigue cracks seen in A. Contralateral condylar stress fracture seen in transverse (C) and dorsal (D) reconstructions supports the reference assessment of *Overt stress fracture from fatigue injury*.^24^, ^26^ Lateral or dorsal to the left.

For condylar stress fracture risk assessment, the reference assessment of *Overt stress fracture from fatigue injury* using sCT and joint surface examination was the same for all five repeats. For DR, two observers also consistently graded the repeats, whilst the assessment of the third observer varied across two levels of risk (Table 2, Figure 2). For sCT, one observer consistently graded the repeats, whilst the assessments of the other two observers varied across two or three levels of risk (Table 2).

### 3.3 Detection of MC3 SBI using DR

Observer 1 did not assess image sets from three horses because image quality was felt to affect diagnostic interpretation. Similarly, Observer 4 did not assess image sets from 11 horses including the horse used for the repeated image set to evaluate intra‐observer variation. Observer 2 and 3 evaluated images for all 31 horses. Consequently, for estimation of sensitivity, specificity, and intra‐class correlation, image sets from 19 of 31 horses were used for detailed analysis.

**FIGURE 3:**
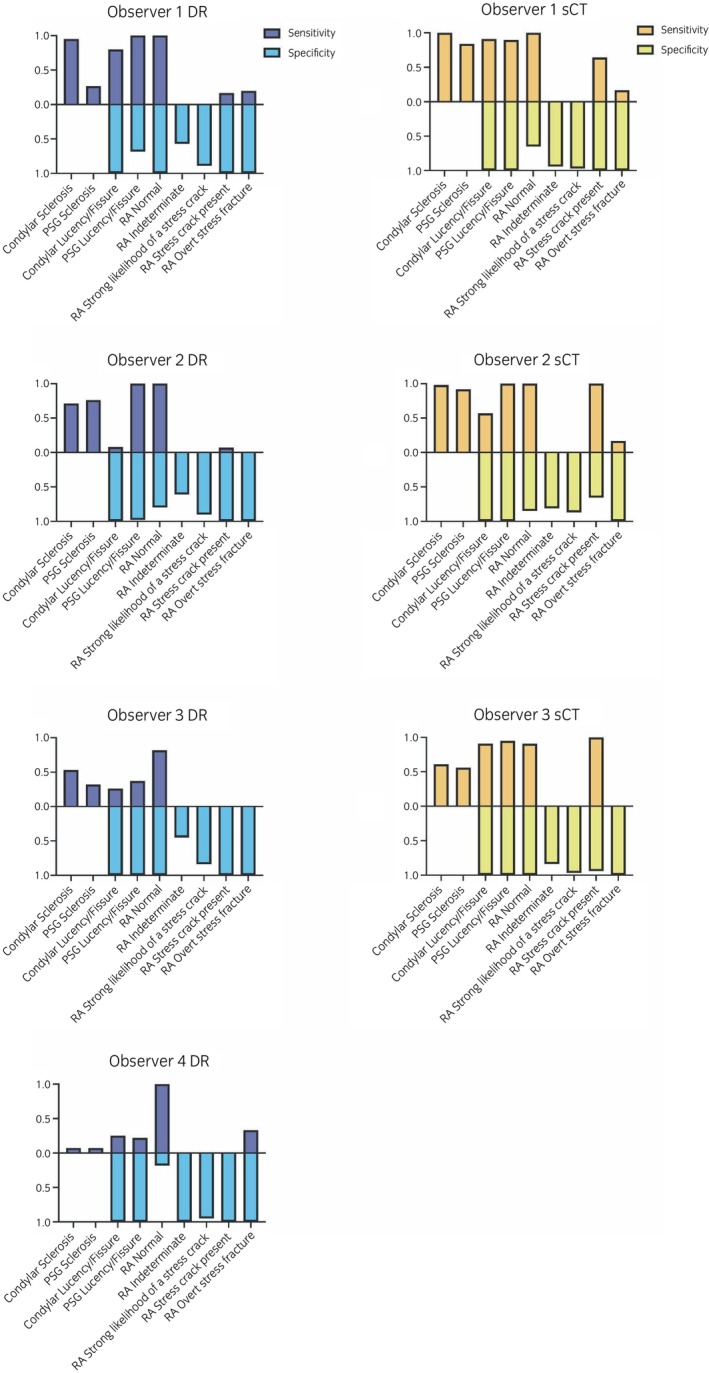
Sensitivity and specificity for detection of joint fatigue injury and condylar stress fracture risk assessment using digital radiography (DR) and standing computed tomography (sCT). Sensitivity was not reported for the *Indeterminate* and *Strong likelihood of a stress crack* categories as the reference assessment did not record any horses in these categories. Specificity could not be estimated for detection of condylar or parasagittal groove (PSG) sclerosis as the gold standard reference assessment was always yes for the detection of these changes. RA, risk assessment.

Significant differences from the reference assessment of the extent of dense subchondral bone across the observers were most evident for bones with absent or mild changes (File S2: Figure S3, File S3). Specificity was above 65% for detection of PSG lucency and 100% for the detection of condylar lucency for all observers (Figure 3). Sensitivity for detection of structural changes in subchondral bone was variable between observers (Figure 3). Observers 3 and 4 had low sensitivity for detection of PSG lucency/fissure.

### 3.4 Detection of MC3 SBI using sCT

The three observers that evaluated sCT images assessed image sets from all 31 horses. Twenty‐one percent of the observations (10/48) differed significantly from the reference assessment of the extent of dense subchondral bone across the observers were identified (File S3). Significant differences were most often identified for assessment of the extent of dense PSG subchondral bone with absent or mild change (File S3). Specificity for detection of PSG and condylar lucency was 100%. Sensitivity for detection of PSG or condylar lucency was lower than specificity across the observers, except for detection of PSG lucency by Observer 2 (Figure 3).

Observer 1 did not measure the PSG subchondral plate thickness in one horse. Some bias from the reference measurements were identified, particularly for Observers 1 and 3 (File S2: Figure S4). Both positive and negative bias amongst the observers was identified.

**FIGURE 4:**
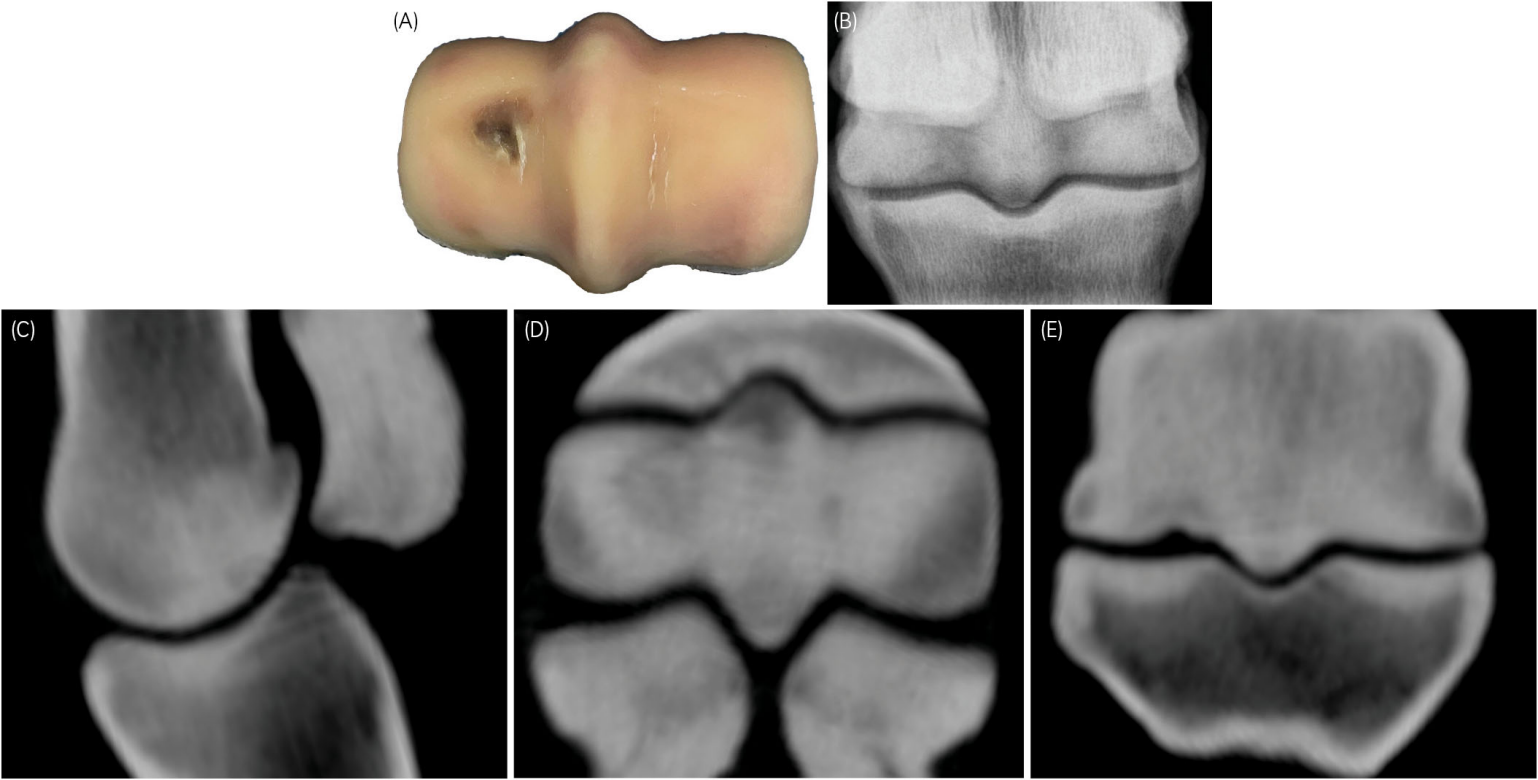
Postmortem (A), digital radiography (DR) (B), and standing computing tomography (sCT) images (C–E) from a racing Thoroughbred with severe subchondral changes (Horse no. 6, Figure 1). A large lateral palmar osteochondral disease (POD) lesion spanning the condyle and PSG together with a medial PSG subchondral lucency is evident on the sagittal (C), transverse (D) and dorsal (E) sCT, together with sclerosis of the proximal sesamoid bones. Fatigue damage to the third metacarpal joint surface corresponds with the changes evident on visual inspection (A). Subchondral structural changes are also evident with flexed dorsopalmar radiography (B) but are more difficult to identify. The reference assessment was *Overt stress fracture from fatigue injury*. A lower risk assessment rating was provided by three of four observers interpreting DR imaging. Concordance with the reference assessment was improved with sCT imaging. Lateral or dorsal to the left. Biaxial mid‐body proximal sesamoid bone stress fracture was present in the contralateral fetlock.

For measurement of sagittal plane PSG subchondral lucency area, data points were excluded if they spanned the PSG and the condyle or were present only in the condyle (Figure 4). All observers demonstrated close agreement with the reference assessment for the presence of a lucency (File S2: Table S3). Some bias from the reference measurements was also observed for measurement of PSG subchondral lucency area in the sagittal plane. Measurements by Observers 1 and 3 showed positive bias from the reference measurements (File S2: Figure S5). Measurement bias was also more variable than estimates of PSG subchondral plate thickness.

### 3.5 Reliability of detection of MC3 SBI using DR and sCT

**FIGURE 5:**
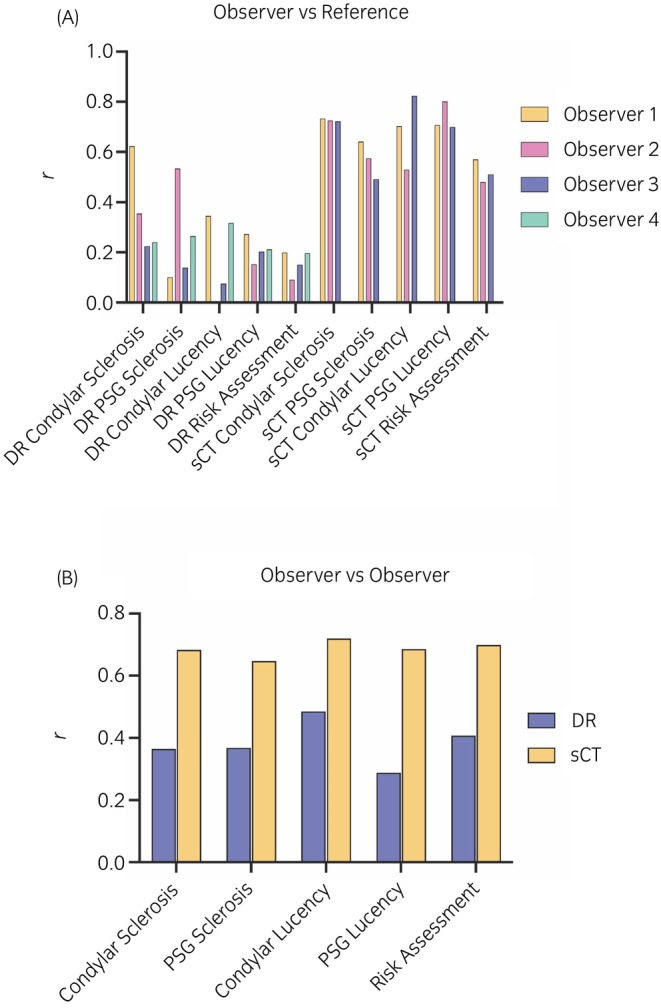
Agreement between diagnostic imaging observers estimated by the intraclass correlation coefficient statistic yield higher correlation coefficients for sCT compared with DR for both detection of structural change in the subchondral bone and for condylar stress fracture risk assessment. (A) Agreement between observer estimation and the gold standard reference. (B) Agreement between observer estimations.

For agreement with the reference measurements, the range of *r* for DR assessment was 0.0011–0.62 across all categories. Correlation coefficients were generally below 0.5 (Figure 5A). With DR, Observer 1 achieved the highest overall agreement with the reference assessment and Observer 3 achieved the lowest overall (Figure 5A). The range of *r* for sCT was 0.49–0.82 (Figure 5A). Correlation coefficients were generally between 0.50 and 0.75. With sCT, Observer 1 was the most consistent across the assessment variables. For agreement between observers the range of *r* for DR was 0.29–0.48 and for sCT was 0.65–0.72 (Figure 5B).

### 3.6 Accuracy of risk assessment for PSG subchondral fatigue injury with DR and sCT

For the horse used for assessment of intra‐observer repeatability, the category of *Indeterminate* was most often assigned from the DR image sets (Table 2, Figure 2) different from the reference assessment of *Overt stress fracture from fatigue injury*. In contrast, a risk assessment of *Stress crack present from subchondral fatigue injury* was most often assigned from sCT for this horse (Table 2). Contralateral condylar stress fracture was present at the time of euthanasia of this horse.

Sensitivity was not reported for the *Indeterminate* and *Strong likelihood of a stress crack* categories as the reference assessment did not record any horses in these categories (Tables 3 and 4). For horses with a standard level of risk rated as *Normal*, observer risk assessment often agreed with the reference assessment. Specificity for risk assessment was much higher than sensitivity for all observers (Figure 3). The only category with 100% sensitivity with DR was *Normal* for two observers. For horses with elevated risk assessed to have a *stress crack present from subchondral fatigue injury* or an *overt stress fracture from fatigue injury* observers generally underestimated the level of risk. Horses with elevated risk were most accurately assessed by Observer 4 with associated higher diagnostic sensitivity (Table 3, Figure 3). For each observer, diagnostic sensitivity of risk assessment was improved with sCT imaging, particularly for horses with elevated risk of injury.

**TABLE 3:** Accuracy of condylar stress fracture risk assessment in the racing Thoroughbred using digital radiography.

Risk category	Reference	Observer 1	Observer 2	Observer 3	Observer 4
Normal	9	9	13	9	17
Indeterminate		9	6	9	
Strong likelihood of stress crack from subchondral fatigue injury		1		1	1
Stress crack present from subchondral fatigue injury	7				
Overt stress fracture from fatigue injury	3				1

*Note*: Analysis was based on the 19 horses that all four observers assessed. The reference assessment was based on evaluation of sCT images combined with pathological assessment of the articular surface of the distal end of the third metacarpal bone. For the three horses with a reference assessment of *overt stress fracture from fatigue injury*, condylar stress fracture was present in the contralateral fetlock of two of these horses. In the remaining horse, biaxial mid‐body proximal sesamoid bone stress fracture was present. Observer 4 also rated the horse with contralateral biaxial mid‐body proximal sesamoid bone stress fracture similarly.

**TABLE 4:** Accuracy of condylar stress fracture risk assessment in the racing Thoroughbred using standing computed tomography.

Risk category	Reference	Observer 1	Observer 2	Observer 3
Normal	11	18	15	10
Indeterminate		2	6	5
Strong likelihood of stress crack from subchondral fatigue injury		1	4	1
Stress crack present from subchondral fatigue injury	14	9	5	15
Overt stress fracture from fatigue injury	6	1	1	

*Note*: Analysis was based on all 31 horses that all three observers assessed. The reference assessment was based on evaluation of sCT images combined with pathological assessment of the articular surface of the distal end of the third metacarpal bone. For the six horses given a reference assessment of *overt stress fracture from fatigue injury*, condylar stress fracture was present in the contralateral fetlock of four of these horses, with biaxial mid‐body proximal sesamoid bone stress fracture in another horse. In the remaining horse, the contralateral limb was not available to the study. Observers 1 and 2 also rated the horse with contralateral biaxial mid‐body proximal sesamoid bone stress fracture similarly.

For DR, three horses were given a reference assessment of *overt stress fracture from fatigue injury*. Condylar stress fracture was present in the contralateral fetlock of two of these horses. In the remaining horse, biaxial mid‐body proximal sesamoid bone stress fracture was present. Observer 4 also rated the horse with contralateral biaxial mid‐body proximal sesamoid bone stress fracture similarly.

For sCT, six horses were given a reference assessment of *overt stress fracture from fatigue injury*, which included the three horses rated as such in the DR data set. In this set of six horses, condylar stress fracture was present in the contralateral fetlock of four of these horses, with biaxial mid‐body proximal sesamoid bone stress fracture in another horse. Fracture of the first phalanx was also present on one of the joints with condylar fracture, and one horse with contralateral condylar fracture also had an associated open fetlock luxation. In the remaining horse, the contralateral limb was not available to the study. Observers 1 and 2 rated the horse with contralateral biaxial mid‐body proximal sesamoid bone stress fracture similarly.

Regarding agreement with the reference analysis, the range of *r* was 0.09–0.20 for DR assessment and 0.48–0.57 for sCT (Figure 5). For agreement between readers, *r* = 0.41 for DR and *r* = 0.70 for sCT (Figure 5 and File S2: Figure S6).

## 4 DISCUSSION

In this report, we evaluated use of DR and sCT imaging for injury prevention screening for condylar stress fracture. The limbs studied had MC3 PSG and condylar SBI with a range of severity from absent to severe that reasonably reflects Thoroughbred racehorse populations racing and training across the world. With DR imaging, there were often significant differences in assessment between observer and reference assessments of the extent of the dense subchondral bone, suggesting that it is more difficult for different observers to consistently identify mild to moderate adaptive change. Some observers had low sensitivity for detection of PSG subchondral fissure/lucency, suggesting assessment of the severity of the PSG lucency/fissure is also difficult with DR imaging. A similar trend was also found with sCT imaging but to a lesser degree. These conclusions were also supported by the overall low diagnostic sensitivity for detection of PSG subchondral sclerosis and PSG lucency/fissure with DR, which was improved with sCT.

Condylar stress fracture risk has been linked to subchondral plate functional adaptation by bone modelling,^27^, ^40^ to increase the bone volume fraction and the thickness of the subchondral plate to better resist the high cyclic loads associated with racing. We found some bias in measurement of this variable amongst observers, suggesting that small differences in subchondral plate thickness are challenging to interpret clinically, particularly between observers. This might have been partially caused by measuring the thickness at slightly different reconstructed planes between observers. Like subchondral plate thickness, mechanical function has been linked to the presence of PSG subchondral lucency and its area in the sagittal plane.^29^, ^30^ With sCT, there was good agreement between the observers and the reference assessment regarding whether there was a subchondral PSG lucency to measure. However, observer estimation of sagittal area was variable, and bias was identified between observer and reference measurements. This variation suggests that the usefulness of lucency sagittal area for risk assessment screening^29^ may be limited and that a more objective sCT‐based risk assessment is needed.

Visual evaluation of the MC3 distal joint surface from eight horses after cartilage digestion led to change in the risk assessment because a small amount of macroscopic fatigue damage was observed in the postmortem joint surface photographs but was not detectable because of the resolution of the clinical sCT. Findings were similar between horses. Most were also assessed as having normal risk by the panel of observers and it is acknowledged that the mildness of the changes may mean that their risk was low. The finding that not all horses with stress cracks arising from SBI in this study were detectable using either sCT or DR also suggests that serial assessment could be an important consideration in racehorse screening to detect SBI sooner rather than later before a horse with low risk of serious injury develops a progressive SBI associated with much greater risk. The experience of the authors suggests that SBI lesions and their associated risk can change rapidly over time.

Contralateral PSG subchondral lucencies and associated fatigue injury are known to be associated with catastrophically injured horses.^27^, ^28^ More data are needed from a larger number of horses to better define this association. The Thoroughbred used for assessment of intra‐observer variation had contralateral condylar stress fracture at the time of euthanasia and the study limb was assessed as high risk with an overt stress fracture from fatigue injury that merited clinical intervention from the sCT evaluation that was then affirmed by the joint surface evaluation. We found that PSG lucency/fissure in this horse was inconsistently identified using DR amongst the observers and more consistently identified using sCT. One observer did not identify lucency/fissure in any of the repeats. For risk assessment, none of the observers rated any of the repeat image sets from this horse with the highest level of risk. Experimental mechanical testing data that includes this bone connects structural changes in subchondral fetlock bone to mechanical compromise,^29^, ^30^ increasing confidence in the gold standard risk assessments in this study. To better inform clinicians regarding risk assessment for condylar stress fracture, the image sets used in this study have been made available via the Dryad data repository (datadryad.org) as a training set for veterinarians interested in fetlock risk assessment as part of injury prevention in racing Thoroughbreds.

For agreement with the reference assessment of MC3 subchondral structural changes, we found that the reliability of DR was generally poor (*r* < 0.5). For sCT reliability was generally moderate (*r* ≥ 0.50 to <0.75). Similar reliabilities were found when observer assessments were compared with each other. Whilst sCT assessments were generally more reliable, observer training may be important to ensure that any screening assessments are as accurate as possible.

Observers were generally better at detecting and assessing structural changes than risk assessment for imminent injury. Diagnostic sensitivity was lower than specificity for all observers and both imaging methods. We found diagnostic sensitivity for risk assessment was improved for all three observers who assessed both types of imaging when assessments were performed from sCT imaging. For risk assessment with DR, three horses received a reference assessment of overt stress fracture from fatigue injury and all these animals had contralateral fetlock stress fracture. Only one of these horses was rated by one of four observers as having the highest level of risk. With sCT, five of the six horses with a similar reference assessment had contralateral fetlock stress fracture. Only one of these horses was rated by two observers as having the highest level of risk. These findings support the reference assessment of *high risk of imminent injury needing rehabilitation or surgery* given current published data, albeit from a relatively small number of horses,^27^, ^28^ and suggest that observers generally underestimated imminent risk of serious injury. Whilst advanced imaging, such as sCT, has the potential to improve identification of at‐risk horses, more data connecting structural change with mechanical compromise of the joint surface are needed to inform interpretation of diagnostic images.^30^, ^41^

There were several limitations to this study. Assessment of fetlock diagnostic imaging is subjective and variable. The risk assessment approach described in Table 1 was developed from the expertise of the authors for the purpose of investigating sensitivity and specificity of assessments of both structural changes and associated serious injury risk in an ex vivo study. Whilst the high incidence of contralateral stress fractures provides initial validation,^27^, ^28^ further prospective study of the risk assessment scale is needed in a longitudinal clinical study using a larger number of horses to confirm the utility of the risk assessment approach used in the present study. However, in an in vivo study, allowing horses assessed as having a high risk of imminent serious injury to continue to participate in racing and monitoring these animals to see if a stress fracture develops is not ethical and would not be viewed favourably by society. Mechanical assessments of such horses using sCT and validated 3D finite element modelling to assess mechanical compromise should help to confirm connections between structural compromise in the affected bone and specific features evident on diagnostic imaging relevant to risk assessment.^42^ Alternatively, comparison with historic data at the same racetrack may also help with validation. For example, use of sCT imaging for screening of individual horses is currently being used by Racing Victoria (www.racingvictora.com.au) for the Spring Racing Carnival, particularly for horses racing in the Melbourne Cup race, which has historically been associated with a high risk of injury. Our study design has estimated this variation to better understand clinical usefulness of DR and sCT for fetlock screening. DICOM file sets were anonymised using the Horos DICOM viewer. One observer used Osirix for image viewing, which inadvertently grouped the repeated blinded image sets together, reducing blinding. This problem was addressed by reading the image sets as independently as possible. The library of samples we used appropriately represented the range of MC3 subchondral structural changes found clinically. However, the image set may not cover all potential clinical features or range of lesion severities. In this regard, intra‐observer repeatability was only performed with one horse with PSG SBI to avoid making the data set used for analysis very large and reducing observer compliance. Intra‐observer repeatability with different levels of subchondral bone structural change may be different. The presence of proximal sesamoid bone fracture in three limbs made evaluation of the MC3 subchondral bone more difficult, particularly one horse with biaxial comminuted mid‐body displaced fractures. Such specimens were not excluded because the distal MC3 contained important subchondral pathology relevant to the study's aims. Two of the four observers did not assess DR images from some of the horses because of concerns about image quality regarding resolution of the trabecular patterns in the MC3 distal metaphysis, and it was considered that the diagnostic quality of the DR images was of a generally lower level than that of best‐practice in vivo imaging. Positioning of isolated limbs for flexed DP imaging is challenging relative to in vivo radiography. Furthermore, storage at −20°C and associated changes in the soft tissue compliance may also have affected image quality in this study. To address this problem, estimation of diagnostic sensitivity and specificity was limited to the horses that were assessed by all four observers, reducing sample size. In contrast, in vivo sCT imaging may be associated with more motion artefact with a consequent reduction in image quality, particularly if imaging is performed with the limb in a non‐weight bearing position. Depending on the type of sCT system used clinically, imaging may be performed in a natural weight‐bearing standing stance^26^ or with the limb suspended by ropes.^43^ Extension of this research approach to an in vivo image set is necessary to further assess DR image sets using optimal radiographic technique and sCT image sets obtained in the live horse. At UW‐Madison, clinical experience with the Asto CT Equina for fetlock imaging suggests that there is little difference between ex vivo and in vivo imaging with this system as sCT images are made with the horse standing square with a natural stance. Finally, reference assessment of subchondral sclerosis was limited to analysis of sCT image data. These assessments could have been strengthened by objective assessment of the sclerotic volume in the MC3/MT3 bone end. Development of such a method is a current research priority.

In conclusion, we describe diagnostic sensitivity and specificity for detection of MC3 subchondral fatigue injury in the fetlock in an ex vivo model. Development of PSG SBI is a consequence of the high cyclic loads inherent to current Thoroughbred training and racing practices. Subchondral MC3/MT3 fatigue microdamage accumulates with age and cumulative race starts^34^ raising the possibility of targeted screening of horses in high‐risk subgroups. Identifying when microdamage has reached a critical level requires more research and specific criteria for interpretation of subtle diagnostic imaging changes,^6^, ^10^, ^27^, ^28^, ^30^, ^40^ to generate more data for full clinical validation of risk assessment levels for screening. Risk assessment through screening with diagnostic imaging is a promising approach to improve injury prevention in racing Thoroughbreds. However, when diagnostic imaging is used for risk assessment as opposed to clinical diagnosis, the potential discrepancy between the presence of a subchondral stress crack identifiable pathologically by joint surface observation, and the typical assessment by diagnostic imaging through identification of a cortical discontinuity, fissure or overt fracture line is a challenge that needs more research and more observer training. Knowledge of sensitivity and specificity of fetlock lesion detection by DR and sCT is critical information needed to develop improved screening programs for racehorses and to begin to establish appropriate roles for DR and sCT imaging in injury prevention in Thoroughbreds. Our results show improved detection of MC3 subchondral structural change and risk assessment for condylar stress fracture with sCT in an ex vivo model and contribute to improved understanding about how diagnostic imaging may best be used to detect SBI that may predispose to serious or catastrophic condylar stress fracture. Further longitudinal clinical study using risk assessment from diagnostic imaging appears warranted. Such work would generate important new data on healing of PSG SBI and inform management strategies such as rest or surgical treatment with a transcondylar bone screw.

## AUTHOR CONTRIBUTIONS

**Soroush Irandoust:** Methodology; software; data curation; investigation; formal analysis; supervision; project administration; writing – review and editing; visualization. **Linnea M. O'Neil:** Methodology; investigation; formal analysis; visualization; writing – review and editing. **Christina M. Stevenson:** Investigation. **Faith M. Franseen:** Investigation. **Pieter H. L. Ramzan:** Conceptualization; methodology; investigation; writing – review and editing. **Sarah E. Powell:** Investigation; writing – review and editing. **Sabrina H. Brounts:** Investigation; writing – review and editing; methodology; funding acquisition. **Samantha J. Loeber:** Investigation; writing – review and editing. **David L. Ergun:** Investigation; resources. **R. Chris Whitton:** Conceptualization; writing – review and editing; funding acquisition. **Corinne R. Henak:** Investigation; writing – review and editing; project administration; supervision; funding acquisition. **Peter Muir:** Conceptualization; methodology; data curation; investigation; supervision; funding acquisition; project administration; resources; writing – original draft; writing – review and editing; formal analysis; visualization.

## FUNDING INFORMATION

Grayson‐Jockey Club Research Foundation.

## CONFLICT OF INTEREST STATEMENT

P. Muir is a Founder of Asto CT, a subsidiary of Centaur Health Holdings Inc. and a Founder of Eclipse Consulting LLC. S. H. Brounts is a clinical advisor to Asto CT. D. L. Ergun is the Chief Executive Officer of Asto CT.

## DATA INTEGRITY STATEMENT

Dr. Muir and Dr. Irandoust had full access to all of the data in the study and take responsibility for the integrity of the data and accuracy of the data analysis.

## ETHICAL ANIMAL RESEARCH

Research ethics committee oversight not currently required by this journal: the study was performed on material obtained from a disposal service.

## INFORMED CONSENT

Not applicable: equine samples were sourced via a disposal service.

### PEER REVIEW

The peer review history for this article is available at https://www.webofscience.com/api/gateway/wos/peer-review/10.1111/evj.14131.

## ANTIMICROBIAL STEWARDSHIP POLICY

Not applicable.

## Supporting information


**Supplementary File S1.** Fetlock imaging assessment form template.

**Supplementary File S2.** Supplementary figures and tables.

**Supplementary File S3.** Supporting data.

## Data Availability

The data that support the findings of this study are openly available in an online data set from the Dryad Data Repository at https://doi.org/10.5061/dryad.dncjsxm55.
