# Supplementary material for: Comparison of radiography and computed tomography for identification of third metacarpal structural change and associated assessment of condylar stress fracture risk in Thoroughbred racehorses
Source: Equine Vet J. 2024 Aug 14;57(3):723–36. doi: 10.1111/evj.14131 (PMC11982434; doi:10.1111/evj.14131)
Supplement: Supplementary file 2 — Supplementary File S2. Supplementary figures and tables. [file EVJ-57-723-s001.pdf]

Equine Veterinary Journal BEVA

Figure S1: Distribution of joint pathology scores from assessment of articular cartilage and subchondral bone from the distal end of MC3 in Thoroughbred racehorses. Damage to the cartilage and subchondral bone ranged from absent to severe in the sample population. LC – lateral condyle, LPSG – lateral parasagittal groove, MPSG – medial parasagittal groove, MC – medial condyle.

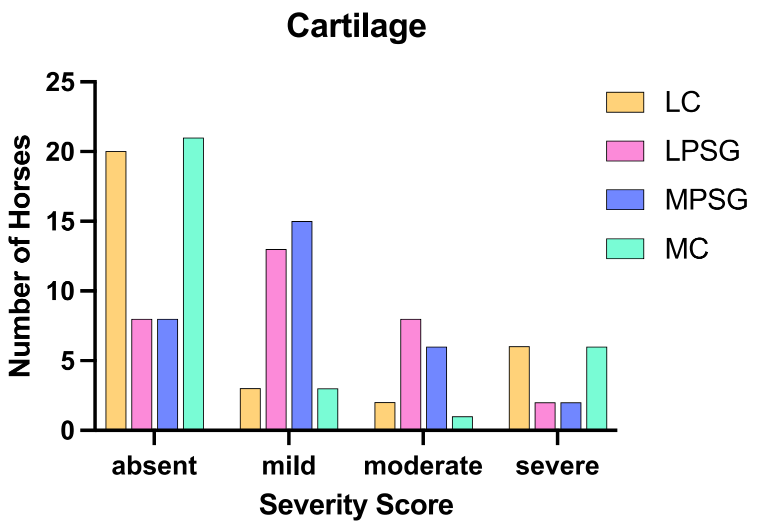

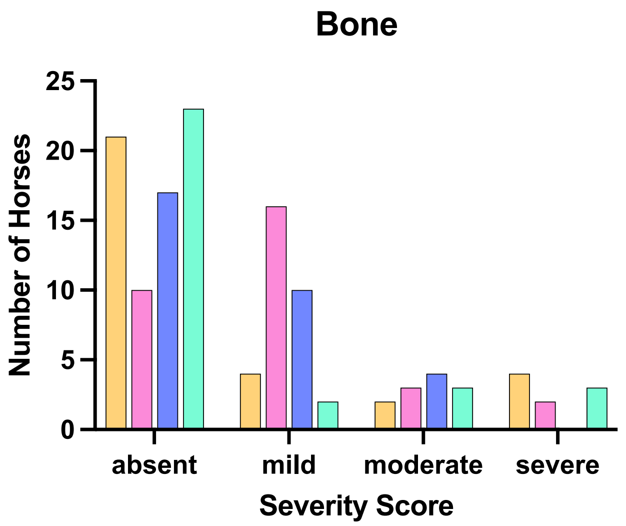

Equine Veterinary Journal BEVA

Figure S2: Image montage presenting the surface appearance of the subchondral bone plate of the distal end of each third metacarpal bone after digestion of the articular cartilage for the 31 racing Thoroughbreds studied. **Note**. Lateral is to the left and palmar to the bottom.

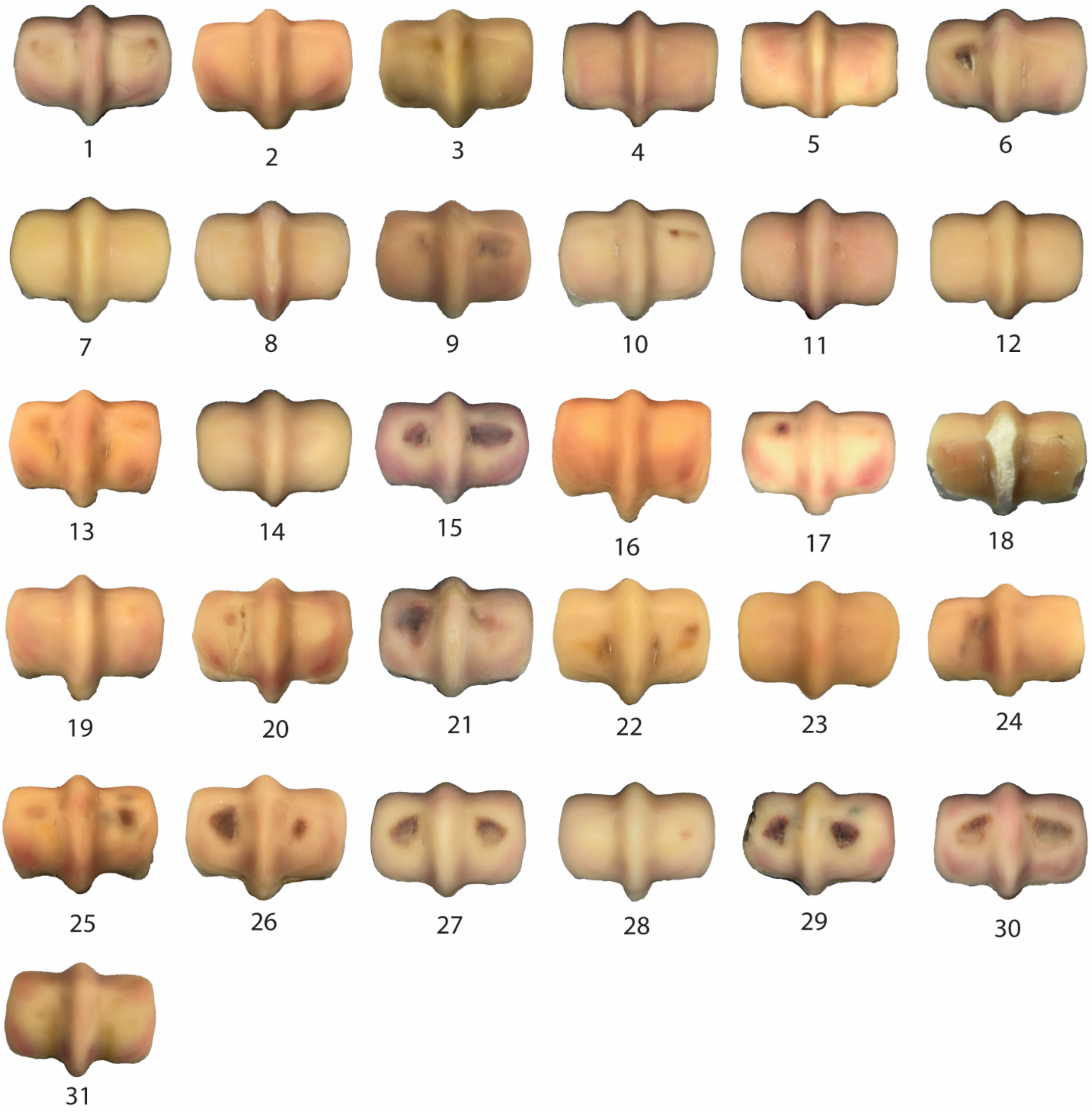

Figure S3: Postmortem (**A**), digital radiography (DR) (**B**), and standing computing tomography (sCT) images (**C-E**) from a racing Thoroughbred with mild subchondral injury (Horse #23, Figure 1). A very small subchondral defect is evident in the lateral PSG that was not detectable by DR or sCT. Little modelling of the subchondral plate is evident radiographically. The reference assessment was *Stress crack* while observers interpreting sCT imaging provided an assessment of *Normal*. **Note**. Lateral or dorsal to the left.

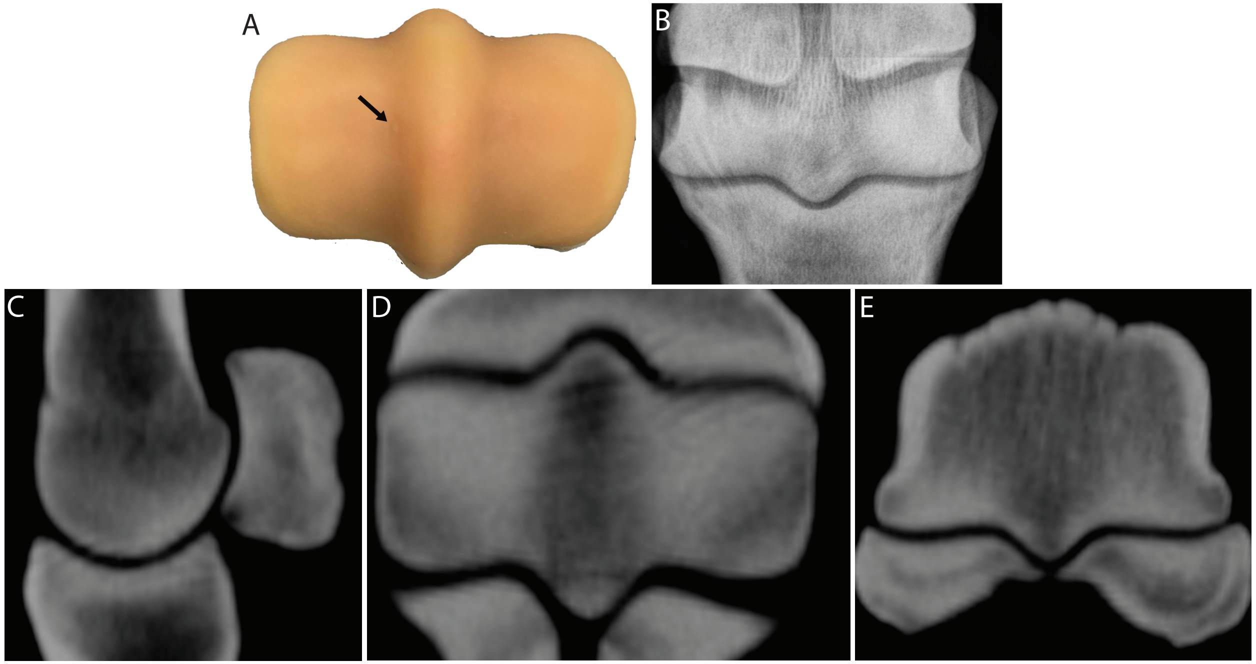

Equine Veterinary Journal BEVA

Figure S4: Bland-Altman plots for measurement of parasagittal groove subchondral plate thickness from standing computed tomography imaging in Thoroughbred racehorses. Measurements by the observers showed positive (Observer 1) or negative bias (Observer 3) from the reference measurements.

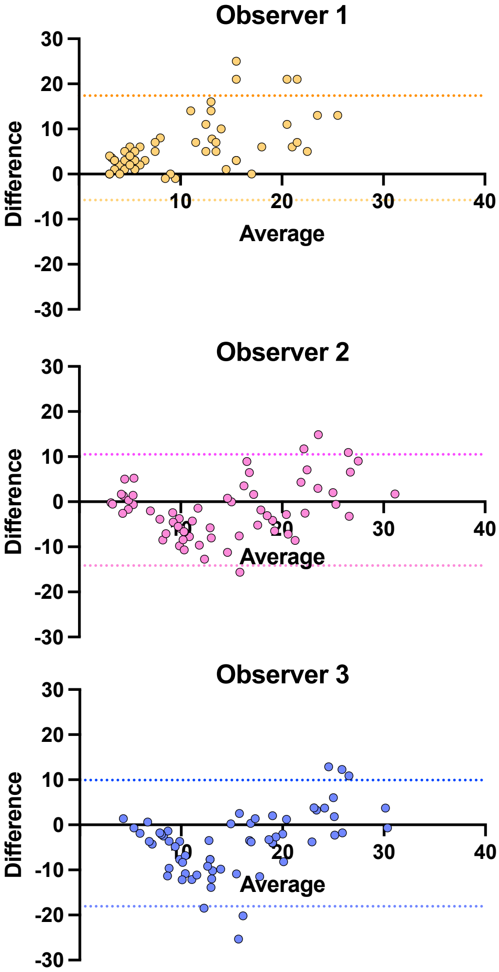

Equine Veterinary Journal BEVA

Figure S5: Bland-Altman plots for measurement of parasagittal groove subchondral lucency area (mm^2) in the sagittal plane from standing computed tomography imaging in Thoroughbred racehorses. Measurements by Observers 1 and 3 showed positive bias from the reference measurements. The data points included in the plot were those with measurements from both the observer and the reference assessment. This resulted in a sample size of 14, 16, and 9 for Observers 1 to 3, respectively.

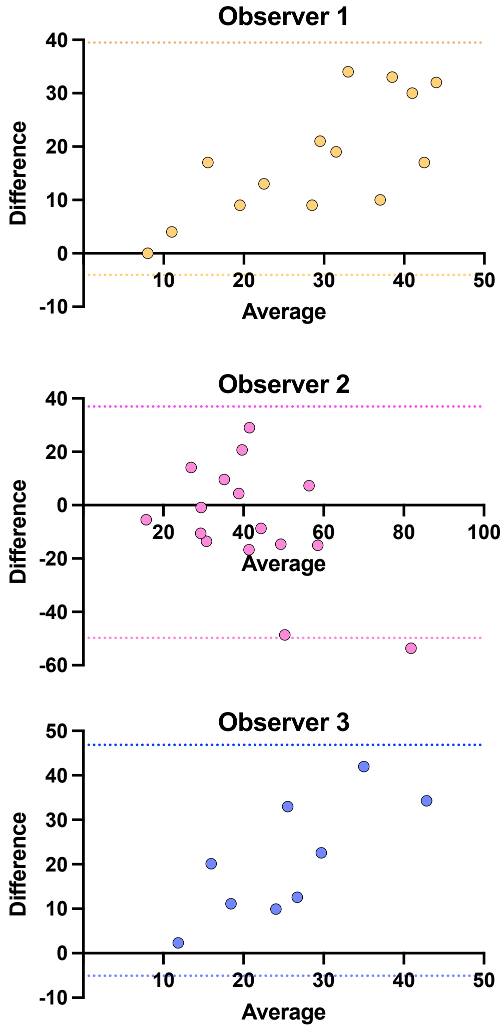

Equine Veterinary Journal

BEVA

Figure S6: Postmortem (**A**), digital radiography (DR) (**B**), and standing computing tomography (sCT) images (**C-E**) from a racing Thoroughbred with a lateral parasagittal groove (PSG) focal subchondral bone lucency because of fatigue injury (Horse #10, Figure 1). The lucency seen on sagittal (C), transverse (D), and dorsal (E) sCT reconstructions correspond to the linear PSG subchondral fatigue cracks seen in A. The reference assessment was *Overt stress fracture from fatigue injury.* A lower risk assessment rating was provided by all 4 observers interpreting DR imaging. Concordance with the reference assessment was improved with sCT imaging. **Note**. Lateral or dorsal to the left. Condylar stress fracture was present in the contralateral fetlock.

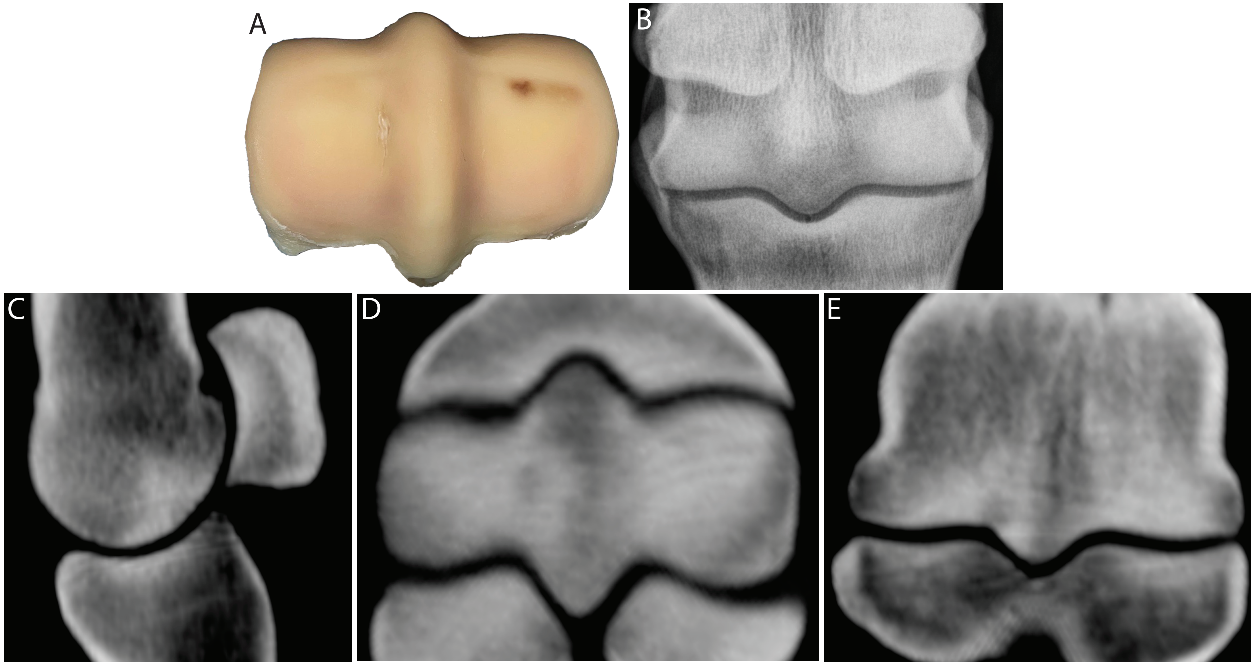

Table S1: Intra-observer repeatability in detection of structural change reported by digital radiography for the racing Thoroughbred with five blinded repeats of the image set.

Observer	Extent of dense metacarpal subchondral bone				Focal lucency/fissure			
	LC	LPSG	MPSG	MC	LC	LPSG	MPSG	MC
1	1 (1, 2)	1 (0, 1)	0 (0, 1)	2 (1, 2)	0 (0)	1 (1, 2)	1 (1)	0 (0)
2	1 (0, 2)	1 (1, 2)	2 (1, 2)	1 (1, 2)	0 (0)	0 (0, 1)	1 (1, 2)	0 (0)
3	1 (1)	1 (0, 1)	0 (0, 1)	1 (1)	0 (0)	0 (0)	0 (0)	0 (0, 1)

Note. Data are reported as median (range). Observer 4 reported poor image quality for all 5 of the DR image sets and did not score any of them. The extent of dense subchondral bone was evaluated as 0 – absent, 1 – mild, 2 – moderate, 3 – severe and focal lucency/fissure was evaluated as 0 – absent, 1 – poorly defined, no surrounding increased density, 2 – poorly defined with surrounding increased density, 3 – well defined, no surrounding increased density, 4 – well defined with surrounding increased density (**Figure S1**). LC – lateral condyle, LPSG – lateral parasagittal groove, MPSG – medial parasagittal groove, MC – medial condyle.

Equine Veterinary Journal BEVA

Table S2: Intra-observer repeatability in detection of structural change reported by standing computed tomography for the racing Thoroughbred with five blinded repeats of the image set.

Observer	Extent of dense metacarpal subchondral bone				Subchondral plate thickness (mm)		Focal lucency/fissure				Focal lucency parasagittal area (mm^2)	
	LC	LPSG	MPSG	MC	LPSG	MPSG	LC	LPSG	MPSG	MC	LPSG	MPSG
Reference	1 (1, 2)	2 (1, 2)	2 (2)	1 (1, 2)	17±2.35	19±1.30	0 (0)	4 (4)	3 (3)	0 (0)	24 (21, 28)	56 (52, 61)
1	2 (2, 3)	2 (1, 2)	2 (1, 2)	2 (2, 3)	9±1.10	9±1.14	0 (0, 3)	4 (3, 4)	4 (0, 4)	0 (0, 3)	15 (7, 18)	26 (24, 28)
2	2 (2, 3)	2 (2, 3)	2 (2, 3)	2 (2, 3)	21±1.29	21±2.54	0 (0)	4 (4)	3 (1, 4)	0 (0)	38 (28, 44)	27 (19, 36)
3	2 (2)	2 (2, 3)	2 (2, 3)	2 (2)	21±3.27	21±4.86	0 (0)	4 (4)	4 (4)	0 (0)	13 (11, 14)	14 (11, 18)

Note. Data are reported as median (range) or mean±standard deviation. The extent of dense subchondral bone was evaluated as 0 - absent, 1 - mild, 2 - moderate, 3 - severe and focal lucency/fissure was evaluated as 0 - absent, 1 - poorly defined, no surrounding increased density, 2 - poorly defined with surrounding increased density, 3 – well defined, no surrounding increased density, 4 - well defined with surrounding increased density (**Figure S1**). LC - lateral condyle, LPSG – lateral parasagittal groove, MPSG – medial parasagittal groove, MC – medial condyle. Observer 4 did not evaluate sCT image sets.

Equine Veterinary Journal BEVA

Table S3: Observer variation in detection of sagittal plane parasagittal groove subchondral bone lucency in Thoroughbred racehorses using standing computed tomography

		Reference Assessment	
		Yes	No
**Observer 1**	Yes	14	2
	No	5	40
**Observer 2**	Yes	16	4
	No	3	36
**Observer 3**	Yes	9	5
	No	2	30

Note. The sample sizes for each observer varied because some observers made measurements for lesions that were considered to occupy both the condyle and parasagittal groove regions. For consistency, results are reported for PSG lucencies only. The number of measures for each observer was: Observer 1 - 61, Observer 2 - 59, Observer 3 – 46.
